# Corticospinal excitability in idiopathic normal pressure hydrocephalus: a transcranial magnetic stimulation study

**DOI:** 10.1186/s12987-020-0167-0

**Published:** 2020-02-17

**Authors:** Jani Sirkka, Laura Säisänen, Petro Julkunen, Mervi Könönen, Elisa Kallioniemi, Ville Leinonen, Nils Danner

**Affiliations:** 1 Neurocenter, Neurosurgery, Kuopio University Hospital and University of Eastern Finland, Kuopio, Finland; 2 Department of Clinical Neurophysiology, Kuopio University Hospital, Kuopio, Finland; 3 Department of Applied Physics, University of Eastern Finland, Kuopio, Finland; 4 Department of Clinical Radiology, Kuopio University Hospital, Kuopio, Finland; 5 Department of Psychiatry, University of Texas Southwestern Medical Center, Dallas, USA; 6 Unit of Clinical Neuroscience, Neurosurgery, University of Oulu and Medical Research Center Oulu, Oulu University Hospital, Oulu, Finland

**Keywords:** Idiopathic normal pressure hydrocephalus, Navigated transcranial magnetic stimulation, Lumbar puncture, Corticospinal excitability, Inhibition

## Abstract

**Background:**

Idiopathic normal pressure hydrocephalus (iNPH) is a neurodegenerative disease with an unknown etiology. Disturbed corticospinal inhibition of the motor cortex has been reported in iNPH and can be evaluated in a noninvasive and painless manner using navigated transcranial magnetic stimulation (nTMS). This is the first study to characterize the immediate impact of cerebrospinal fluid (CSF) drainage on corticospinal excitability.

**Methods:**

Twenty patients with possible or probable iNPH (16 women and 4 men, mean age 74.4 years, range 67–84 years), presenting the classical symptom triad and radiological findings, were evaluated with motor function tests (10-m walk test, Grooved Pegboard and Box & Block test) and nTMS (silent period, SP, resting motor threshold, RMT and input–output curve, IO-curve). Evaluations were performed at baseline and repeated immediately after CSF drainage via lumbar puncture.

**Results:**

At baseline, iNPH patients presented shorter SPs (p < 0.001) and lower RMTs (p < 0.001) as compared to normative values. Positive correlation was detected between SP duration and Box & Block test (rho = 0.64, p = 0.002) in iNPH patients. CSF drainage led to an enhancement in gait velocity (p = 0.002) and a steeper IO-curve slope (p = 0.049).

**Conclusions:**

Shorter SPs and lower RMTs in iNPH suggest impaired corticospinal inhibition and corticospinal hyperexcitability. The steeper IO-slope in patients who improve their gait velocity after CSF drainage may indicate a higher recovery potential. Corticospinal excitability correlated with the motor function of the upper limbs implying that the disturbance in motor performance in iNPH extends beyond the classically reported gait impairment.

## Background

Idiopathic normal pressure hydrocephalus (iNPH) is a neurodegenerative disease with prevalence increasing with age [1, 2]. Conventionally, the symptoms of iNPH have been characterized with the Hakim´s triad which includes cognitive decline, urinary incontinence and gait disturbance [1]. Brain imaging typically shows enlargement of the cerebral ventricles and obliteration of parasagittal sulci [3]. The pathophysiology behind the symptoms has been attributed to abnormal dynamics of cerebrospinal fluid (CSF). Currently, the only effective treatment is CSF diversion by shunt surgery. However, for unclear reasons, shunt surgery does not offer alleviation of the symptoms in all patients. Depending on patient selection, less than 2/3 improve their functional status while some patients experience only a subjective benefit. This emphasizes the need to identify biomarkers for shunt surgery responders in iNPH [4, 5].

While the gait dysfunction is the most prominent as well as the most treatment responsive symptom of iNPH, other disease-related motor dysfunctions have not been investigated as thoroughly. Recent studies have suggested that impaired balance may contribute to the gait dysfunction in iNPH [6, 7]. Furthermore, it has also been suggested that iNPH might affect upper limb function presenting as hypokinesia, which shares several features with the motor dysfunctions of Parkinson’s disease [8]. However, the precise neuronal mechanisms that underlie the symptoms of iNPH remain unknown. Previous studies have shown that the corticospinal tract functions normally but the functional connectivity of central movement-regulating mechanisms may be disturbed [9–11].

In this study, we aimed to characterize the excitability of the motor cortex and the corticospinal tract in iNPH. For this purpose, transcranial magnetic stimulation (TMS) offers a non-invasive and painless research tool [12, 13] and until now, only three TMS studies have been carried out in iNPH [10, 11, 14]. The results have pointed towards abnormal corticospinal inhibition. However, in the previous studies, TMS was performed without navigation, for which reason the results could be postulated to derive from disease-related anatomical abnormalities. In iNPH patients ventriculomegaly and obliteration of cortical sulci may influence the effect of TMS, which is determined by the distance from the stimulation coil to the cortex [3, 15]. In the current study, we evaluated the corticospinal excitability using a navigated TMS (nTMS) system, which enables magnetic resonance image (MRI) guided targeting and increases the spatial accuracy of the stimulation [15]. Furthermore, longitudinal examinations within subjects retain their comparability in nTMS [16].

The specific aims of the current study were (1) to characterize the disease related changes of corticospinal excitability in iNPH, (2) to evaluate associations between corticospinal excitability and clinical symptoms of iNPH, and (3) to assess the immediate impact of CSF drainage on corticospinal excitability in both upper and lower limbs using nTMS.

## Methods

### Subjects

Twenty patients were recruited for this study from an ongoing prospective iNPH study at Kuopio University Hospital (KUH) (Table 1). All patients had previously undergone a neurological evaluation and were referred to KUH for surgical evaluation due to suspected iNPH. The patients had probable or possible iNPH determined by the classical symptom triad [1] and brain imaging [3]. Other neurological comorbidities were regarded as exclusion criteria [3]. The patients were required not to use any medications with effects on TMS responses such as antidepressants or antipsychotics [17]. A score of at least 20 points in the Mini-Mental State Examination (MMSE) [18] was required to ensure fluent cooperation during the study and maintenance of attention throughout the TMS session, since TMS responses may be affected by the level of attention [19]. All patients were checked to be eligible for both TMS [20] and MRI [21]. The study protocol was approved by the local research ethics committee (274/2016). All participants gave written informed consent. The patients did not receive any financial compensation for their participation.

**Table 1:** Demographics and clinical characteristics of the iNPH patients

Gender (n)	
Male	4 (20%)
Female	16 (80%)
Age (years)	
Mean ± SD	74.4 ± 4.1
Range	67–84
MMSE (points)	
Mean ± SD	26.4 ± 3.2
Range	20–30
Evans index	
Mean ± SD	0.38 ± 0.04
Range	0.33–0.52
Callosal angle (degrees)	
Mean ± SD	62.6 ± 16.8
Range	33.5–111.1
DESH (n)	3 (15%)
Comorbidities (n)	
Hypertension	12 (60%)
Coronary artery disease	9 (45%)
Dyslipidemia	9 (45%)
Adult-onset diabetes	5 (25%)
B12 vitamin deficiency	3 (15%)
Arthrosis	3 (15%)
Hypothyroidism	3 (15%)

*DESH* disproportionately enlarged subarachnoid space [22], *MMS* mini mental state examination

A control population for the TMS responses was gathered from a pre-existing TMS database of KUH from two previous studies with healthy subjects [23, 24] in which nTMS was performed using an NBS version 2.2.0 (Nexstim Plc, Helsinki, Finland) system. In total 20 cases were selected based on age and gender (10 women and 10 men, mean age 69.2 years ± 7.1 years, range 60–81).

### Flow of the study

**Fig. 1:**
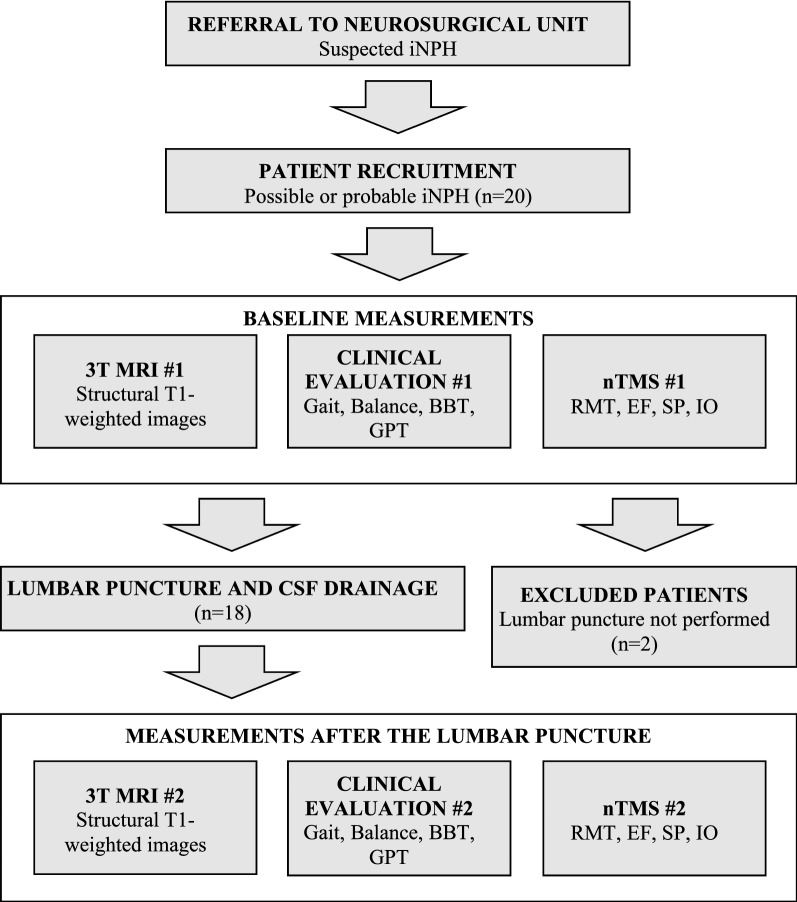
Flow chart of the study. 3T MRI, 3 Tesla magnetic resonance imaging; *SP* silent period, *RMT* resting motor threshold, *EF* electric field, *IO*  input–output curve, *BBT*  Box and Block Test, *GPT* Grooved Pegboard Test, *CSF* cerebrospinal fluid

The flow of the study is presented in Fig. 1. Each patient underwent the clinical assessments as well as nTMS and MRI studies twice, 1 day prior to the lumbar puncture (LP) and on the day of the LP. The clinical assessments were repeated immediately after the LP and were followed by nTMS and MRI within 4 to 6 h.

### Clinical assessment

At baseline, patients were evaluated using three domains of the iNPH scale: gait, neuropsychology and balance [25]. Gait was assessed in accordance with the iNPH scale with the total score of gait (TSG) in a 10-m walking test including ordinal rating, gait velocity and number of steps [25]. Balance was scored in accordance with the scale [25]. We applied a modified neuropsychology domain consisting of two tests, the Grooved Pegboard Test (GPT) (Lafayette Instruments, Lafayette, IN) and CERAD [26]. The GPT was used to evaluate fine motor function of upper limbs and CERAD to evaluate overall level of cognitive functions. In addition to the modified iNPH scale, the Box and Block Test (BBT) [27] was used to evaluate gross motor function of the upper limbs. In the TAP test (TT) [28, 29], CSF was drained up to 40 ml via LP and all tests, with the exception of CERAD, were repeated identically as before the drainage (Fig. 1).

### Radiological imaging

All subjects underwent clinical diagnostic MRI with a 3T MRI scanner (Philips Achieva, The Netherlands). The images were evaluated by an experienced neuroradiologist to exclude subjects with other neurological disorders than iNPH. Structural 3D T1-weighted images (1 mm × 1 mm × 1 mm voxel size) were used for nTMS. Evans index [30] and callosal angle [31] were manually determined from the MR-images (Table 1).

### Navigated transcranial magnetic stimulation

NTMS was performed using an NBS system (version 4.3.1, Nexstim Plc, Helsinki, Finland) and a figure-of-eight coil with biphasic pulse waveform. The nTMS protocol was performed identically before and after the LP. The primary motor cortex (M1) of the left hemisphere was stimulated and continuous electromyography (EMG) was recorded to register TMS-induced motor evoked potentials (MEPs) from the contralateral limb muscles with an integrated EMG system. When stimulating the M1 at the representation area of the upper limb, responses were registered from the first dorsal interosseous (FDI), the abductor pollicis brevis and the extensor carpi radialis muscles. When stimulating the cortical representation area of the lower limb, responses were registered from the abductor hallucis brevis (AHB), the tibialis anterior and the vastus lateralis muscles. MEPs from FDI to AHB were analyzed and other muscles were used as controls to detect and avoid concurrent muscle activation. A peak-to-peak amplitude of 50 µV was the approval limit of MEPs. All responses with EMG-activity (above 50 µV peak-to-peak amplitude) 50 ms before the stimulus were excluded from the analysis [32].

The nTMS protocol abided with the IFCN guidelines [33]. First, M1 was mapped in the vicinity of the hand knob to determine the hotspot (stimulation target) inducing MEPs with highest amplitude in the FDI muscle. At the stimulation target, resting motor threshold (RMT), silent period (SP) and input–output curve (IO) were measured (Table 2). Corticospinal excitability was evaluated as stimulator output percentage (% of maximum stimulator output, %-MSO) and with the calculated electric field (EF) strength (V/m). The RMT was measured at the stimulation target using an integrated iterative threshold assessment tool [34, 35]. When the RMT is measured as %-MSO it is influenced by the coil-to-cortex distance and may therefore theoretically be influenced by the CSF drainage. Therefore we used the calculated EF at the stimulation target as an additional measure of corticospinal excitability in order to eliminate the effect of possible anatomical alterations [16, 24, 36, 37]. The SP is a stimulation induced rest in voluntary muscle activation and its duration is considered to reflect the level of corticospinal inhibition [38]. The SP was calculated as an average duration of five trials induced by stimulating at 120% of the RMT [39]. The IO measurement was performed using a sequence of 160 stimulations with varying stimulation intensities (80–150% of the RMT in 10% steps) and intervals (3–5 s) in a randomized order. A Boltzmann sigmoidal function was used to determine the maximum value (IO-max), the mid-point of the curve (IO-50) and the slope of the curve (IO-slope) [40]. After completing all measurements in the upper limb, the M1 was mapped for the representation area of the contralateral lower limb to determine the hotspot of the AHB. The RMT and EF were determined in a similar manner as for the FDI in the upper limb.

**Table 2:** Transcranial magnetic stimulation (TMS) responses measured in this study

	Definition	Neuronal background	Interpretation	References
Motor threshold	–	Glutamatergic system	High motor threshold means decreased corticospinal excitability	[33, 41, 42]
Resting motor threshold (RMT)	Corticospinal excitability (% of maximum stimulator output)	–	–	–
Electric field (EF)	Corticospinal excitability (induced electric field, V/m, at the cortex)	–	–	–
Silent period (SP)	Corticospinal inhibition	GABAergic system	Long silent period means increased corticospinal inhibition	[33, 38, 43]
Input–output curve (IO-slope)	Dose-dependent response to TMS	Synaptic connectivity and plasticity	The steepness of the IO-slope reflects the level of synaptic connectivity and plasticity	[40, 44–46]

*GABA* gamma-aminobutyric acid

### Statistical analysis

The normality of distributions of each parameter was assessed with the Kolmogorov–Smirnov and Shapiro–Wilk tests. Non-parametric tests were used for all measures, since the majority were not normally distributed. Spearman’s test was used to determine correlations between different measures in the iNPH patients. The iNPH group was divided into two subgroups based on the TT response: TT+ : at least 10% improvement in gait velocity in the TAP test and TT−: under 10% improvement in gait velocity in the TAP test. TMS responses were compared between the iNPH and the control group (RMT/EF and SP) as well as between the divided iNPH subgroups using the independent-samples Mann–Whitney U test. Measures with repeated measurements before and after the LP were statistically compared in pre- and post-LP situations with the Wilcoxon signed-rank test. P-values < 0.05 were considered as statistically significant. Statistical analyses were performed with SPSS (version 24.0; IBM Corporation, Somers, NY).

## Results

**Fig. 2:**
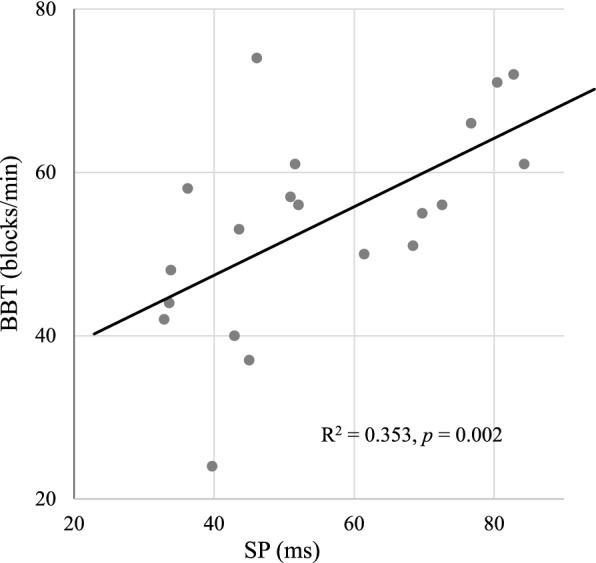
Correlation between Box and Block test (BBT) and silent period (SP) duration at baseline

Twenty patients were evaluated for the study, two of which were excluded (LP could not be performed, n = 1, and the symptoms were regarded as too mild to pursue with invasive testing, n = 1) after the baseline measurement (Fig. 1). At baseline (Table 3), the RMTs of upper and lower limbs were significantly lower in the iNPH patients as compared with the control group (upper limb: U = 11.5, p < 0.001; lower limb: U = 47.0, p < 0.001). Similarly, the cortical EF strength for the upper limbs was higher in the iNPH group (U = 79.5, p = 0.001). The iNPH group had also a significantly shorter SP duration than the control group (U = 73.0, p < 0.001). In the iNPH group the RMTs of the upper and lower limbs correlated positively with each other (rho = 0.56, p = 0.010) and the SP duration correlated positively with the BBT (rho = 0.64, p = 0.002, Fig. 2). There were no other correlations between TMS responses and motor parameters (Table 4).

**Table 3:** Silent period durations (SPs) and resting motor thresholds (RMTs) in iNPH patients and healthy controls

	iNPH (n = 20) mean ± SD, range	Control (n = 20) mean ± SD, range	*p*-value
Upper limb SP (ms)	55.3 ± 17.7, 32.9–84.3	78.7 ± 16.1, 58.3–113.0	< 0.001
Upper limb RMT (%-MSO)	26.0 ± 4.4, 18.0–36.0	45.4 ± 12.2, 29.0–71.0	< 0.001
(V/m)	81.1 ± 13.0, 61.0–103.0	103.6 ± 23.6, 62.0–152.0	0.001
Lower limb RMT (%-MSO)	41.5 ± 7.6, 27.0–60.0	59.2 ± 13.9, 37.0–84.0	< 0.001

Significant differences are reported according to the Mann–Whitney U test for independent samples

*MSO* maximum stimulator output

**Table 4:** Baseline correlations between TMS responses, neuropsychologic performance and motor parameters in iNPH patients

	TSG	Gait velocity	Steps	BBT	GPT^a^	CERAD
Upper limb SP	0.327	0.331	− 0.305	0.639^*^	− 0.311	0.095
Upper limb RMT	− 0.021	− 0.070	0.147	0.298	− 0.389	− 0.212
Upper limb EF	− 0.130	− 0.170	0.065	0.064	− 0.266	− 0.176
Lower limb RMT	− 0.043	− 0.098	0.058	0.089	− 0.185	− 0.147
Lower limb EF	− 0.208	− 0.139	0.084	− 0.007	0.058	− 0.059

n = 20

*SP* silent period, *RMT* resting motor threshold, *EF* electric field, TSG total score of gait, *BBT* Box and Block Test, *GPT* Grooved Pegboard Test

*Indicates significant correlation (*p* = 0.002, Spearman’s rho)

^a^One outlier excluded from statistical analyzes

**Fig. 3:**
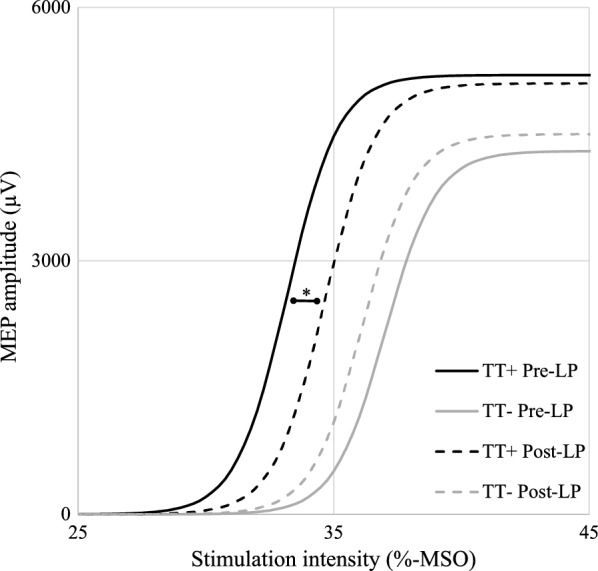
Input–output curves before and after lumbar puncture with cerebrospinal fluid (CSF) drainage. The slope of the input–output curve steepened in the TT+ subgroup after CSF drainage. *Significant difference, p = 0.028, Wilcoxon signed-rank test. *MEP* motor evoked potential, *MSO* maximum stimulator output. *Pre-LP* before lumbar puncture, *Post-LP* after lumbar puncture, *TT+* patients who improved at least 10% in the TAP test, *TT*− patients who improved under 10% in the TAP test

As a result of the LP and CSF drainage the iNPH patients showed a significant increase in TSG and gait velocity likewise increased step length reflected as a decrease in the number of steps. From TMS responses the IO-slope exhibited a significant increase after the LP (Table 5). The study population was divided into two nine-person subgroups based on the TT response with a predefined cut point of 10% improvement in gait velocity (TT+ and TT−). Gait velocity was significantly slower in the TT+ subgroup as compared to the TT− subgroup in the pre-LP situation (p = 0.040). After the LP there were no significant differences in the motor parameters between the subgroups. The upper limb EF was statistically lower in the TT+ subgroup than in the TT− subgroup in the post-LP situation (p = 0.024) but no significant within-subgroup changes were detected in the EF (Table 6). The IO-slope exhibited a significant increase after the LP in the TT+ subgroup (p = 0.028, Fig. 3), but not in the TT− subgroup (p > 0.999).

**Table 5:** TMS responses and motor parameters before and after lumbar puncture

	Pre-LP mean ± SD	Post-LP mean ± SD	*p*-value
Upper limb SP (ms)	55.5 ± 17.4	54.1 ± 20.1	0.227
Upper limb RMT (%-MSO)	26.4 ± 4.3	26.1 ± 3.9	0.462
Upper limb EF (V/m)	82.1 ± 13.0	83.9 ± 16.0	0.601
Lower limb RMT (%-MSO)	42.2 ± 7.6	43.1 ± 8.3	0.146
Lower limb EF (V/m)	149.3 ± 44.2	139.2 ± 34.8	0.586
IO-max (mV)	4.8 ± 2.7	4.9 ± 2.8	0.959
IO-50 (%)	35.8 ± 5.7	36.4 ± 4.9	0.642
IO-slope	2.6 ± 1.2	3.1 ± 1.4	0.049*
TSG (score)	53.7 ± 25.0	58.0 ± 25.2	0.039*
Gait velocity (m/s)	0.86 ± 0.27	0.95 ± 0.25	0.002*
Steps (number)	23.1 ± 6.7	21.7 ± 4.9	0.017*
BBT (pcs)	54.5 ± 12.5	54.6 ± 10.2	0.634
GPT (s)	142.3 ± 105.2	125.2 ± 54.0	0.705
Balance (score)	62.5 ± 12.4	66.1 ± 4.0	0.141

n = 18

*SP* silent period, *RMT* resting motor threshold, *EF* electric field, *IO-max* the maximum value of the input–output curve, *IO-50* the mid-point of the input–output curve, *IO-slope* the slope of the input–output curve, *TSG* total score of gait, *BBT* Box and Block Test, *GPT* Grooved Pegboard Test, *Pre-LP* before lumbar puncture, *Post-LP* after lumbar puncture

* Significant differences (*p *< 0.05) have been indicated (Wilcoxon signed-rank test)

**Table 6:** TMS responses and motor parameters before and after lumbar puncture in divided subgroups

	Pre-LP			Post-LP				
	TT+ mean ± SD	TT− mean ± SD	*p*-value^a^	TT+ mean ± SD	TT− mean ± SD	*p*-value^a^	TT+^b^ *p*-value	TT−^b^ *p*-value
Upper limb SP (ms)	51.6 ± 18.6	62.2 ± 16.5	0.161	48.0 ± 23.3	61.0 ± 15.6	0.093	0.110	0.779
Upper limb RMT (%-MSO)	25.3 ± 4.4	27.6 ± 4.2	0.436	24.6 ± 3.4	27.7 ± 3.9	0.077	0.287	0.831
Upper limb EF (V/m)	80.1 ± 14.3	84.1 ± 12.1	0.489	76.4 ± 16.1	91.3 ± 12.5	0.024*	0.407	0.075
Lower limb RMT (%-MSO)	42.3 ± 8.4	42.0 ± 7.3	0.387	43.1 ± 8.0	43.0 ± 9.1	0.436	0.388	0.252
Lower limb EF (V/m)	158.7 ± 47.4	139.9 ± 41.3	0.387	138.4 ± 32.1	140.0 ± 39.2	0.931	0.327	0.767
IO-max (mV)	5.2 ± 2.9	4.3 ± 2.5	0.470	5.1 ± 3.3	4.5 ± 2.3	1.000	0.767	0.735
IO-50 (%-MSO)	34.1 ± 3.2	38.0 ± 7.6	0.299	35.9 ± 3.8	37.1 ± 6.3	0.873	0.173	0.310
IO-slope	2.5 ± 1.2	2.7 ± 1.3	0.837	3.4 ± 1.6	2.6 ± 1.0	0.470	0.028*	1.000
TSG (score)	42.8 ± 21.4	64.6 ± 24.5	0.077	51.9 ± 25.8	64.2 ± 24.4	0.340	0.018*	0.674
Gait velocity (m/s)	0.74 ± 0.24	0.99 ± 0.24	0.040*	0.90 ± 0.24	1.01 ± 0.26	0.340	0.008*	0.314
Steps (number)	25.8 ± 7.5	20.5 ± 4.6	0.077	22.8 ± 5.5	20.6 ± 4.2	0.340	0.011*	0.575
BBT (pcs)	49.4 ± 14.2	59.6 ± 8.6	0.113	50.7 ± 11.2	58.6 ± 7.8	0.136	0.952	0.438
GPT (s)	111.1 ± 22.4^c^	125.7 ± 43.7	0.481^c^	109.9 ± 26.9^c^	117.1 ± 21.2	0.321^c^	0.889^c^	0.779

n = 18

*SP* silent period, *RMT* resting motor threshold, *EF* electric field, *IO-max* the maximum value of the input–output curve, *IO-50* the mid-point of the input–output curve, *IO-slope* the slope of the input–output curve, *TSG* total score of gait, *BBT* Box and Block Test, *GPT* Grooved Pegboard Test, *Pre-LP* before lumbar puncture, *Post-LP* after lumbar puncture, *TT+* patients who improved at least 10% in the TAP test, *TT−* patients who improved under 10% in the TAP test

* Indicates significant differences (*p* < 0.05)

^a^Comparison of between-group differences before and after the LP (Independent-Samples Mann–Whitney U test)

^b^Comparison of within-group differences before and after the LP (Wilcoxon signed-rank test)

^c^One outlier excluded from statistical analyzes

## Discussion

This is the first iNPH study to characterize the effect of CSF drainage on corticospinal excitability using nTMS. The results showed disturbed inhibition and hyperexcitability in the corticospinal pathways of both upper and lower limbs. As a novel TMS finding in iNPH, we found a significant steepening of the IO-slope in patients who had improved gait performance in the tap test.

The characteristic imaging findings of iNPH may have a substantial impact on TMS-responses due to disease-related anatomical alterations affecting the distance from the stimulation coil to the cortex, which alters the effect of TMS according to electromagnetic principles [3, 37]. Thus, previous TMS studies without imaging-based navigation must be interpreted with some caution. One previous study with manually targeted TMS suggested that iNPH patients have decreased RMTs of lower limbs, whereas another study did not find similar results in upper limbs [10, 11]. In the present study, we paid attention to possible anatomical variations affecting TMS responses and used nTMS with continuous visualization of the stimulation target based on individual MRI anatomy. Nevertheless, in accordance with the previously mentioned non-navigated study, our results showed lowered RMTs in the lower limbs of iNPH patients as compared to the healthy control group [10]. Furthermore, abnormally low RMTs were observed also in the upper limbs of the iNPH patients and the RMTs of upper and lower limbs correlated with each other. The decreased RMT in the upper limb is a novel finding in iNPH and likely reflects a more generalized dysfunctions of the motor pathways as previously suggested in TMS studies and supports the clinical reports on the involvement of the upper limbs [8, 10]. In line with the RMT, the EF of the upper limb was also significantly lower in the iNPH group which supports the findings of increased corticospinal excitability, since the EF calculation takes into account stimulator-dependent differences as well as disease-related anatomical alterations affecting the distance from the stimulation coil to the cortex [16, 24]. Previous studies have shown that lower RMTs are associated with a thinning of M1 in healthy subjects which might partly influence our finding, since there are also reports of cortical thinning in iNPH [47, 48]. Furthermore, in previous studies, lowered RMTs have been hypothesized to derive from increased activity of glutamatergic system [41, 42]. Accordingly, disturbances in glutamatergic system might also underlie the hyperexcitability of corticospinal pathways in iNPH observed in the current study. Interestingly, a recent TMS study indicated that a disturbance in cortical inhibitory cholinergic circuits is related to the gait impairment in iNPH which supports the pathophysiological role of the M1 [11].

Our results also showed shortened SPs in iNPH patients which was in line with previous reports [10, 14]. The duration of the SP has been attributed to cortical inhibitory gamma-aminobutyric acid (GABA) interneurons, mostly GABA_B_ [33, 43]. From this perspective, GABAergic mechanisms may also play a role in iNPH resulting in reduced inhibition in corticospinal pathways. The SP duration did not correlate with the RMT, which supports the view that there are multiple contributing mechanisms behind the motor dysfunctions in iNPH. In the current study, corticospinal inhibition measured by SP correlated positively with upper limb gross motor function (BBT). The SP duration was shorter in patients who performed poorer in the BBT which implies that a more severe impairment in upper limb gross motor function is associated with weaker corticospinal inhibition or vice versa. Unexpectedly, the nTMS responses did not correlate with the GPT or gait measurements. TMS induces a direct activation of the corticospinal pathways, and therefore, it could partially bypass the regulatory premotor cortex and the extrapyramidal tracts including the thalamus and the basal ganglia which all are related to the pathophysiology and the motor symptoms of iNPH [49, 50]. Therefore, the lack of correlation between some TMS responses and the motor parameters may result from a more complex disturbance in the extrapyramidal pathways, which cannot be extensively evaluated by TMS. In agreement with our findings, recent reports have suggested a more widespread motor symptomatology of iNPH with bradykinesia and Parkinson’s like symptoms of the upper limbs and these findings challenge the existing practice of focusing on gait as the only routinely assessed motor function [51, 52].

In this study, we characterized the immediate effect of LP and CSF drainage on corticospinal excitability. Until now, it has not been addressed whether the treatment-induced changes of corticospinal excitability in iNPH are a long-term effect of shunt surgery or if they are mediated by the actual decrease in intracranial pressure. Prior studies have shown shunt surgery to restore corticospinal inhibition and to normalize (elevate) RMT in iNPH patients who achieve substantial clinical benefits [10, 14]. In the present study, patients showed a clinical effect in the TAP test reflected as a significant improvement in TSG, gait velocity and number of steps [28, 29]. However, in the TMS responses no significant changes were observed in SP durations or in RMTs of either upper or lower limb after the LP, whereas an increase in the IO-slope was observed in patients who had a positive tap test outcome (TT+ subgroup). In addition, upper limb EFs between TT+ and TT− subgroups showed a significant difference in the post-LP situation even though no significant change within either subgroup was observed. The IO-slope reflects recruitment of corticospinal output in response to cortical stimulation [44–46]. The steeper IO-slope in patients who improved their gait velocity rapidly after CSF drainage may indicate a higher cortical recovery potential by better preserved synaptic connectivity and plasticity. It might be possible that the previously reported changes in TMS responses after shunt surgery reflect long-term effects of decreased CSF pressure to corticospinal excitability. On the other hand, the IO-slope might be more sensitive in the initial phase after CSF drainage and thus, present an early indicator for the later changes. In diffusion tensor imaging (DTI) studies changes in frontal subcortical white matter tracts, periventricular areas and corpus callosum have been observed after CSF drainage. These DTI findings have been associated with a subsequent clinical improvement and may also underlie the differences in the IO-slopes between the subgroups of the present study [49]. These findings indicate that the background mechanisms of symptom alleviation are more complex than just a mechanic decrease of CSF pressure.

The motor symptoms play a significant role in clinical picture of iNPH but they are only one part of classical symptom triad [1]. The evaluation of cognitive performance and urinary incontinence are beyond the common indications for TMS. The MMSE score as the inclusion criteria is recognized as a limitation of this study. The rationale for requiring a score of 20 or more on the MMSE is based on results of previous research in which the level of attention has been reported to influence TMS parameters [19]. The duration of the TMS protocol was approximately 2 hours during which the patients were required to sit still. Complete muscle relaxation was also required with the exception of the SP evaluation where patients had to activate specific muscle groups on demand. The MMSE score as the inclusion criteria limits the generalization of the results. However, we aimed to recruit a homogeneous study population for this first nTMS study in order to exclude possible confounding factors which might influence TMS parameters. After this pilot study it remains to be verified, whether the results can be generalized in a population which reflects the true clinical variability of iNPH.

## Conclusion

This study showed corticospinal hyperexcitability and impaired corticospinal inhibition in iNPH. The reduced corticospinal inhibition seems to be associated with impaired upper limb motor performance. This finding should be addressed in further studies to evaluate the role of upper limb motor function in the clinical characteristics and pathophysiology of iNPH. At baseline, nTMS could not differentiate patients who benefit from CSF drainage but we observed a steepening of the IO-slope in patients who had improved gait performance in the TT. However, further research is required to determine, whether nTMS can offer a biomarker for predicting outcomes of shunt surgery.

## Data Availability

The datasets used and analyzed during the current study are available from the corresponding author on reasonable request.

## Abbreviations

AHB: abductor hallucis brevis
BBT: Box and Block test
CSF: cerebrospinal fluid
DESH: disproportionately enlarged subarachnoid space
DTI: diffusion tensor imaging
EF: electric field
EMG: electromyography
FDI: first dorsal interosseous
GABA: gamma-aminobutyric acid
GPT: Grooved Pegboard Test
iNPH: idiopathic normal pressure hydrocephalus
IO: input–output curve
LP: lumbar puncture
M1: primary motor cortex
MEP: motor evoked potentials
MMSE: Mini-Mental State Examination
MSO: maximum stimulator output
MRI: magnetic resonance image
nTMS: navigated transcranial magnetic stimulation
RMT: resting motor threshold
SP: silent period
TMS: transcranial magnetic stimulation
TSG: total score of gait
TT: Tap test
